# Elemental mercury vapour poisoning

**DOI:** 10.11604/pamj.2024.49.116.43996

**Published:** 2024-12-10

**Authors:** Ashwin Karnan, Anjana Ledwani

**Affiliations:** 1 Department of Respiratory Medicine, Jawaharlal Nehru Medical College, Datta Meghe Institute of Higher Education and Research, Sawangi (Meghe), Wardha, Maharashtra, India

**Keywords:** Cough, dyspnea, mercury poisoning, respiratory failure

## Image in medicine

A 30-year-old male presented with complaints of cough, chest pain and difficulty in breathing for the past 2 weeks. The patient had no addictions and no known comorbidities. The patient has worked in a chemical laboratory for the past 4 months where they routinely use mercury vapour. Computed tomography (CT) thorax of the patient showed a high attenuation focus of around +2400HU distributed along bilateral lung fields and in both kidneys. Blood and urine showed high levels of mercury. A diagnosis of mercury vapour toxicity was made. The patient was treated with oxygen support, corticosteroids, oral penicillamine, acetylcysteine and intravenous fluids. The patient was counselled about the high possibility of lung fibrosis in the future, discharged and currently is on follow-up. Mercury is a heavy metal used for various purposes available in both organic and inorganic forms. It is commonly used in thermometers, batteries, dental fillings, lamps, and the paint industry, as well as catalysts in the chemical industry. Toxicity depends on the form, dose and rate of exposure. Mercury in all forms impairs cellular function by remodeling protein structures by binding with sulfhydryl groups. It is primarily absorbed through inhaling and very little is absorbed when ingested. The chief target organ is the brain but it can also affect the lungs, kidneys and the skin. Treatment involves cessation of exposure with chelation and supportive therapy. The prognosis is variable and depends on the level of exposure.

**Figure 1:**
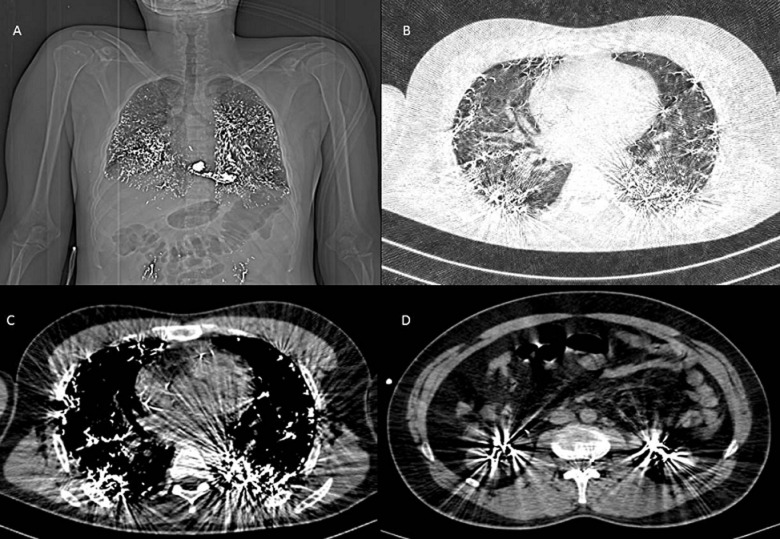
A) bilateral opacities of the patient; B) CT thorax showing high attenuation foci with ground glass opacities in bilateral lung fields; C) CT thorax mediastinal window showing a peripheral distribution with high attenuation focus within right atrium and right ventricle; D) CT abdomen showing high attenuation focus in both kidneys

